# Invisible emotional expressions influence social judgments and pupillary responses of both depressed and non-depressed individuals

**DOI:** 10.3389/fpsyg.2013.00291

**Published:** 2013-05-22

**Authors:** Bruno Laeng, Line Sæther, Terje Holmlund, Catharina E. A. Wang, Knut Waterloo, Martin Eisemann, Marianne Halvorsen

**Affiliations:** ^1^Department of Psychology, University of OsloOslo, Norway; ^2^Department of Neurology, University Hospital of North NorwayTromsø, Norway; ^3^Department of Psychology, University of TromsøTromsø, Norway; ^4^Department of Pediatric Rehabilitation, University Hospital of North NorwayTromsø, Norway

**Keywords:** depression, pupillometry, subliminal perception, facial emotions, face hybrids

## Abstract

We used filtered low spatial frequency images of facial emotional expressions (angry, fearful, happy, sad, or neutral faces) that were blended with a high-frequency image of the same face but with a neutral facial expression, so as to obtain a “hybrid” face image that “masked” the subjective perception of its emotional expression. Participants were categorized in three groups of participants: healthy control participants (*N* = 49), recovered previously depressed (*N* = 79), and currently depressed individuals (*N* = 36), All participants were asked to rate how friendly the person in the picture looked. Simultaneously we recorded, by use of an infrared eye-tracker, their pupillary responses. We expected that depressed individuals (either currently or previously depressed) would show a negative bias and therefore rate the negative emotional faces, albeit the emotions being invisible, as more negative (i.e., less friendly) than the healthy controls would. Similarly, we expected that depressed individuals would overreact to the negative emotions and that this would result in greater dilations of the pupil's diameter than those shown by controls for the same emotions. Although we observed the expected pattern of effects of the hidden emotions on both ratings and pupillary changes, both responses did not differ significantly among the three groups of participants. The implications of this finding are discussed.

## Introduction

Several neuroscience studies show that an extended network involving the medial prefrontal cortex, anterior circulates and amygdala (as well as other anatomically-related limbic, striatal, thalamic, and basal forebrain structures) is dysfunctional in individuals suffering from depression (Price and Drevets, 2011; Stuhrmann et al., 2011). In particular, neuroimaging studies show that the resting metabolism of the amygdala is abnormally elevated and that activations in this neural structure are exaggerated to sad stimuli. However, an opposite hypoactivity of the amygdala has also been observed in some studies, especially with adolescents and children suffering from various psychiatric disorders (e.g., Jones et al., 2009; Brotman et al., 2010; White et al., 2012; but see Yang et al., 2010). Because hyperactivity of the amygdala has been clearly associated with depression in adults, it has been proposed that anti-depressants may exert their therapeutic action by constraining such over-activity (e.g., Murphy et al., 2009; Godlewska et al., 2012).

In addition, at the behavioral level, depressed individuals tend to exhibit a mood-congruent bias, toward negative information (Bradley et al., 1995; Murphy et al., 1999; Murray, 1999; Elliott et al., 2000; Broomfield et al., 2007). For example, depressed individuals remember best negatively-valenced information, show more interference from negative words than positive ones, respond faster to sad than happy words, and prefer to attend to faces showing negative expressions than neutral or positive and tend to interpret ambiguous information as more negative than non-depressed individuals.

The majority of studies that have revealed emotional biases in depressed individuals have used facial emotions as a research tool particularly in neuroimaging research (for a review see Stuhrmann et al., 2011). This research has revealed that “masked” emotional expressions (i.e., not processed consciously) can yield clear changes in behaviour of healthy individuals (Dimberg et al., 2000) as well as result in an exaggerated activity in the amygdala of depressed individuals in response to the implicitly “seen” sad faces and/or blunted responses to happy faces (Suslow et al., 2010; Victor et al., 2010). Some studies have also shown that antidepressant drug treatment can decrease the amygdala's hyperactivity triggered by the non-conscious threat cues (Harmer et al., 2006). Importantly, imaging studies with healthy participants (Vuilleumier et al., 2003) have shown that the amygdala is responsive to face information that is contained within the lowest spatial frequency range (<6 cycles/image) and it is “blind” to other frequencies.

**Figure 1:**
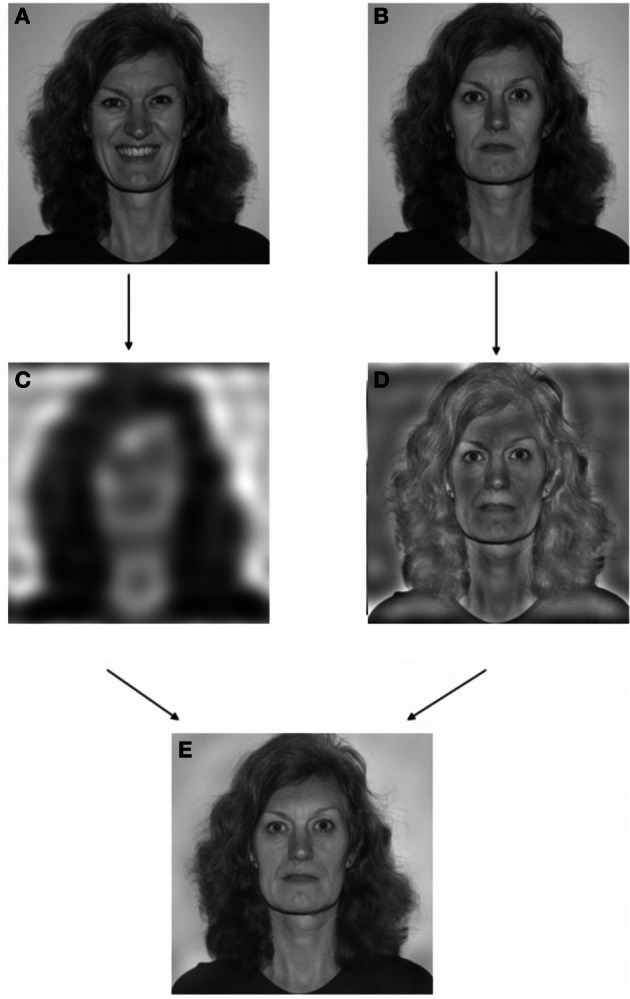
**An example of the editing procedure used to obtain a *hybrid* expressive face.** Image **(A)** and **(B)** are separate photographs of the same individual assuming a “happy” and “neutral” expression, respectively. Image **(C)** is the low-passed version (<6 cycles/image) of Image **(A)**, whereas Image **(D)** is the high-passed version (>7 cycles/image) of Image **(B)**. Image **(E)** is the hybrid picture or a combination of images **(C,D)** with a happy expression embedded exclusively in the lowest spatial frequencies. Nota Bene: images in this illustration are printed at a size smaller than the one used in the actual experiment and the person appearing in this example is not one of the stimulus faces from the Karolinska facial stimuli set and her face was not shown in the experiment.

In a previous study with healthy participants (Laeng et al., 2010) we used face “amalgamations” or face hybrids to induce unconscious processing in healthy individuals of emotional facial expressions that were never consciously recognized as emotional but consistently categorized as “neutral.” Specifically, by using graphic-processing techniques based on Fourier transforms, we merged a filtered low-visual frequency image of an emotional expression (a happy, fearful, or angry face photograph) with a high-frequency image of a neutral facial expression (a neutral face photograph), which masked subjective perception of the low-frequency emotional expression (see Figure 1 for an illustration of the procedure). We garnered evidence that when viewing the amalgamation or “face hybrid”, observers were only aware of seeing the neutral face and the emotional perception was not perceived consciously regardless of focusing attention on the image for several seconds. Such low-pass images preserve rough positions of cheeks and mouth but lose most identifying detail for conscious recognition, whereas the high-pass image preserves details of eyes, nose, mouth, etc. that are most crucial to recognizing identity. Crucially, despite not consciously recognizing that they are looking at an emotional expression, people who viewed the hybrids containing an “unseen” happy low-pass expression nonetheless went on to assign friendlier ratings to the hybrid image. Conversely, if the hybrid's low-pass emotion was angry, people subsequently assigned a less-friendly rating. Laeng and colleagues also showed that the emotion effect became lost in a neurological patient with damage to the left amygdala. This should be expected on the basis that a low-pass emotional expression would have direct ‘low road’ access via the amygdala to trigger a core affective reaction in intact limbic brain systems, independent of conscious perception of the emotional quality of the amalgamated image. That core affective reaction could then influence the evaluations of the face stimulus.

Based on the above findings, we reasoned that (1) if depressed individual's amygdala is sensitized to negative information, including facial expressions, and (2) if the amygdala is responsive to face information that is contained within the lowest spatial frequency range; then showing face hybrids to depressed individuals should result in an exaggerated response to the negative stimuli (e.g., angry or sad emotions in the low spatial frequencies) and, possibly, a blunted response to the positive stimuli (e.g., a happy emotion in the low spatial frequencies).

We collected ratings in social judgements on how “friendly” a series of persons looked with three groups of participants: healthy control participants (*N* = 49), recovered previously depressed (*N* = 79), and currently depressed individuals (*N* = 36). In addition, given that the pupillary response is known to be a valid physiological index of emotional responses and arousal (Bradley et al., 2008) as well as attentional processing in healthy individuals (see for a review Laeng et al., 2012) as well as in depressed individuals (Siegle et al., 2001, 2004), we expected exaggerated pupillary dilations to negative “masked” expressions (anger, fear, and sadness) in the two groups of depression prone individuals compared to the physiological responses of the matched controls to the same stimuli.

## Methods

### Participants

One hundred and fifty-seven subjects participated in the study. A group of participants was categorized comprised as “currently depressed” (*N* = 36), another group as “previously depressed” (*N* = 79), since they had shown recovery from the depressive symptoms, and a final, control, group as “never depressed” individuals (*N* = 42). All diagnoses were based on the Diagnostic and Statistical Manual of Mental Disorders-IV, Text Revision, (DSM-IV-TR; APA, 2000), using the Structured Clinical Interview for DSM-IV, Axis I Disorders (SCID-I; First et al., 1997). Based on the information given in the clinical interview, the subjects were grouped as currently depressed, or having experienced a depressive episode in the past and fully recovered for at least the last 8 weeks or longer, or having never been clinically depressed. Participants meeting criteria for a major depressive episode in partial remission, an on-going or past manic/hypomanic episode, dysthymic disorder, or psychotic symptoms were excluded. Thus, only currently depressed and previously depressed with a history of major depression were included as well as never depressed individuals without any on-going or past Axis I disorders. A history of known brain damage or a major depressive episode (due to a general medical condition) was further exclusion criteria for participation in the study. None of the subjects were hospitalized and they were recruited from a previous study on depression and cognitive vulnerability (Wang et al., 2005, 2006). Some of the depressed individuals and healthy controls were recruited through general practitioners and a local newspaper (Halvorsen et al., 2009, 2011). Vision and hearing were normal or corrected to normal.

The SCID interview was performed by seven interviewers who had been extensively trained by a highly qualified supervisor in its administration. All the interviews were digitally recorded, and 30 of them, 10 from each group, were subsequently randomly sampled for reliability testing. The inter-rater agreement (kappa) between two independent raters per group (never depressed, previously depressed, currently depressed) was 0.9. When the kappa was calculated for rating subjects who had never experienced a depressive episode (i.e., never depressed) and those who had (i.e., previously depressed and currently depressed), the agreement was total, i.e., indicating a highly satisfactory reliability of the group assignments (Halvorsen et al., 2009, 2011).

Participants were examined individually in a quiet and comfortable setting at the Clinic of the Department of Psychology at the University of Tromsø, Norway. They underwent diagnostic assessment, including assessment of severity of depression using the Beck Depression Inventory-II (BDI-II; Beck et al., 1996). The BDI–II is a 21-item self-report inventory designed to assess the presence and severity of depressive symptoms. Each item is rated on a 4-point Likert-type scale ranging from 0 to 3, indicating severity of the symptom. Beck et al. (1996) categorized BDI–II scores as follows: 0–13 minimal, 14–19 mild, 20–28 moderate, and 29–63 severe. A full description of the inventory including psychometric properties can be found in Steer et al. (1999).

The hybrid face experiment took place in the eye lab at the Department of Psychology, University of Tromsø, Norway. In the hybrid face experiment, 26 of the pupil diameter recordings were invalid, and hence we report pupil data for 131 subjects. The loss of pupil diameter recordings for 26 subjects was independent of group membership, age, gender and years of education. The three groups of subjects did not differ significantly concerning gender and years of education. The mean age of “currently depressed” individuals was 37.9 (*SD* = 11.9); of the “previously depressed” 37.3 (*SD* = 9.8), and of the “never depressed” 35.4 (*SD* = 12.7), respectively.

An analysis of variance (ANOVAs) indicated significant group differences on severity of depression (BDI-II total score) and significantly higher scores for “currently depressed” (*M* = 26.31, *SD* = 9.69) compared to “never depressed” (*M* = 2.81, *SD* = 2.99), with “previously depressed” (*M* = 8.06, *SD* = 6.83), scoring in between [*F*_(2, 128)_ = 85.203, *p* < 0.001]. Few individuals were on antidepressant medications at the time of testing (“currently depressed” *N* = 10 or 24% of this group; “previously depressed” *N* = 8 or 9% of this group); which may indicate that a “mild depression” level characterized a majority of the individuals at the time of testing. The regional *medical research ethics committee* (REK) approved this study. All participants gave written informed consent and were paid NOK 150 (€18.80) per hour for their participation.

### Stimuli and apparatus

The face stimuli consisted of 240 gray-scale images selected from the Karolinska Directed Emotional Faces (Lundqvist et al., 1998; Calvo and Lundqvist, 2008). According to a large-scale validation study of the Karolinska Directed Emotional Faces, this included images of 20 male and 20 female models that displayed a neutral expression as well as four emotional expressions (anger, fear, happiness, and sadness). Hybrid faces were created using MatLab® software, according to a method described in Laeng and colleagues (2010) so that emotional facial expressions (anger, fear, happiness, and sadness) were presented only in low spatial frequency (<6 cycles/image) and embedded within neutral facial images of the same model shown in the rest of the bandwidth (>24 cycles/image). This cycles/image of the low-pass versions was chosen as the amygdala has been shown to respond to low spatial frequencies of <6 cycles/image but not to >24 cycles/image (Vuilleumier et al., 2003). As control stimuli, hybrid faces displaying only neutral expressions, and neutral original faces (or neutral broadband images) were used, for a total of 7 image types. All images were presented centred on computer screen and subtended a visual angle of 6.3° (vertical dimension) so as to replicate the viewing conditions of Vuilleumier and colleagues (2003) were replicated. The participant's task was to indicate, on a five point scale, how pleasant/unpleasant it felt to look at each model (one being “most unpleasant” and five ”most pleasant”), and each image remained on screen until the participant responded. The participants were not informed that emotional expressions were embedded in the neutral faces.

## Results

**Figure 2:**
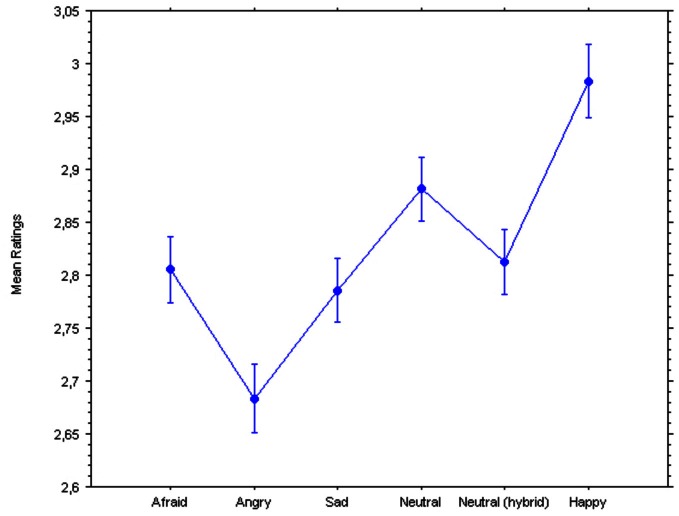
**Ratings of pleasantness of hybrid faces.** Bars represent 95% confidence intervals for within-subject designs (Loftus and Masson, 1994).

**Figure 3:**
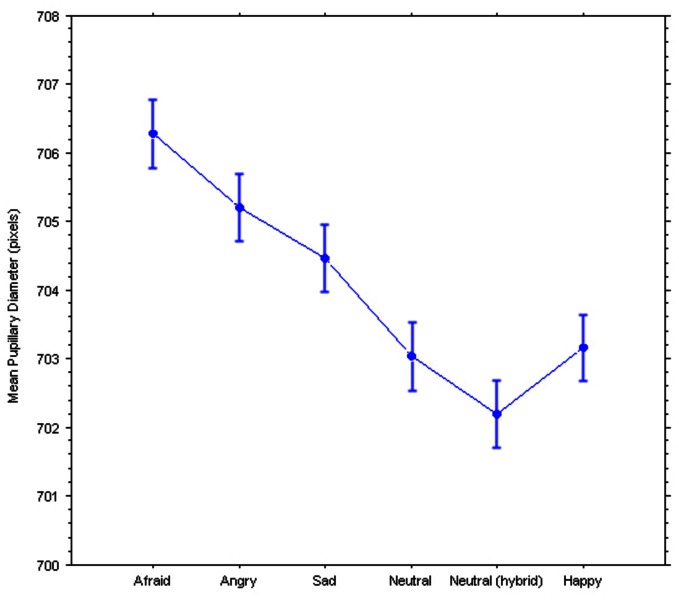
**Pupillary responses to the hybrid faces.** Bars represent 95% confidence intervals for within-subject designs (Loftus and Masson, 1994).

Two repeated-measures ANOVAs were performed on the ratings and the pupillary diameters, with Group (controls, currently depressed, previously depressed) as the between-subject factor and Emotion (afraid, angry, sad, neutral broadband, neutral hybrid, happy) as the within-subject factor, revealed significant main effects of the hidden emotions on both dependent variables (see Figures 2, 3): Ratings, *F*_(2, 5)_ = 53.55, *p* < 0.0001; Pupillary diameters, *F*_(2, 5)_= 2.38, *p* = 0.038. Surprisingly, there were no significant interactions with Group indicating a different response, in either dependent variable, of the controls vs. the currently depressed or the recovered previously depressed individuals: Ratings, *F*_(10, 770)_ = 0.87; Pupillary diameters, *F*_(10, 640)_ = 0.54.

Given that all comparisons between means were within-subject (since there were no between-subjects main effects or interactions), we computed 95% confidence intervals using Loftus and Masson (1994) formula. Hence, the Figures illustrate valid statistical comparisons across means for predicted effects. Negative emotions caused a significant decrease in ratings of friendliness (Figure 1) compared to the neutral and happy expressions. The Newman–Keuls *post-hoc* tests (for significance set at *p* < 0.05) revealed that the Happy expression was significantly different from the following expressions: the Angry expression (critical difference *W*_6_ = 0.047; observed difference = 0.300), the Sad expression (*W*_5_ = 0.045; observed difference = 0.197), the Afraid expression (*W*_4_ = 0.043; observed difference = 0.177), the Neutral Hybrid expression (*W*_3_ = 0.039; observed difference = 0.171), and the Natural Broadband expression (*W*_2_ = 0.033; observed difference = 0.101). Further, the Neutral Broadband expression was significantly different from the following expressions: the Angry expression (*W*_5_ = 0.045; observed difference = 0.199), the Sad expression (*W*_4_ = 0.043; observed difference = 0.096), the Afraid expression (*W*_3_ = 0.039; observed difference = 0.076), and the Neutral Hybrid expression (*W*_2_ = 0.033; observed difference = 0.070). The Neutral Hybrid expression was significantly different from the Angry expression (*W*_4_ = 0.043; observed difference = 0.129). The Afraid expression was significantly different from the Angry expression (*W*_3_ = 0.039; observed difference = 0.123). Finally, the Sad expression was significantly different from the Angry expression (*W*_2_ = 0.033; observed difference = 0.103).

Moreover, negative emotions caused a significant increase in pupil diameter (Figure 2) compared to the neutral and happy expressions. Remarkably, “fearful” faces caused the largest increase in pupil size. Specifically, the Newman–Keuls *post-hoc* tests (for significance set at *p* < 0.05) revealed that the Afraid expression was significantly different from the following expressions: the Neutral Hybrid expression (critical difference *W*_6_ = 2.998; observed difference = 4.098), the Neutral Broadband expression (critical difference *W*_5_ = 2.971; observed difference = 3.245), and the Happy expression (critical difference *W*_4_ = 2.700; observed difference = 3.121). Further, the Angry expression was significantly different from the Neutral Hybrid expression (critical difference *W*_5_ = 2.971; observed difference = 3.014) and the Neutral Broadband expression (critical difference *W*_4_ = 2.700; observed difference = 2.161).

Finally, separate ANOVAs were used to assess the effect of antidepressant use on either ratings or pupillary changes at the time of testing in the two depressed groups, so that both depressive episode (current, previous) and medication (yes, no) were used as between-subjects factors. Both analyses failed to reveal a main effect or interactive effects of antidepressant use.

## Discussion

The present findings are a successful replication of the original findings of Laeng et al. (2010; see also Leknes et al., 2013) regarding changes in social judgments that occur unconsciously as an effect of the low-spatial frequency information that is embedded or “hidden” within the hybrid face stimuli. The pupillary responses also revealed a consistent pattern, where the negative low-passed expressions caused the largest changes in pupillary dilations, with fear and anger causing the largest responses. Previous studies have shown that fearful faces are, in general, very powerful stimuli in activating brain areas (e.g., amygdala, orbito-frontal and prefrontal cortical areas) which are part of the emotional neural network that is dysfunctional in depression (Stuhrmann et al., 2011). Thus, it seems reasonable to conclude that increased pupillary dilation to the expressions of fear and to some extent also anger and sadness do reflect the engagement of the emotional network of the brain.

Surprisingly, the above pattern of results did not differ between the three groups. Such a negative finding in both behavioural (i.e., ratings) and physiological responses (i.e., pupillary changes) between the three groups of participants was unexpected and therefore we can only speculate about the reasons for the lack of a difference in both types of responses. We suggest two possible accounts for the present lack of differences between currently depressed and non-depressed individuals in social judgments of “masked” or invisible facial expressions.

One possible account may be based on the observation that, in major depression, there are reduced levels of norepinephrine (NE) transporters in the locus coeruleus (LC) (Klimek et al., 1997). Given that the LC may be a crucial structure behind the pupillary response to emotional stimuli, a decreased LC response could erase the ability of the pupillary response to expose a negative bias. In fact, the decreased binding of NE transporters is thought to reflect a compensatory downregulation in response to an insufficient availability of NE at the synapse. However, this account, in our opinion, fails to account for the present null findings since we did not find any difference in the behavioral responses or social judgments either, which suggests that a negative bias simply did not occur in these individuals. A variant of this account is the possibility that either the use of antidepressants or psychotherapy itself had succeeded in lowering the level of reactivity of the emotional system to practically baseline level of the controls. Several neuroimaging studies have documented dramatic reduction of the typically hyper-reactive amygdala to emotional stimuli, after a single dose or a moderate use of antidepressants (Murphy et al., 2009; Norbury et al., 2009; Victor et al., 2010; Godlewska et al., 2012) or after a few sessions of psychotherapy (Leichsenring et al., 2004; Linden, 2006; Beutel et al., 2010). We are inclined to exclude the possibility that medications may be behind the present negative findings, since a very small percentage of the depressed individuals were on antidepressants at the time of the study and statistical analyses that included “medication” as a factor failed to reveal any main effects or interactive effects on either ratings or pupillary responses between the subgroups of depressed individuals. In addition, the fact that so few individuals were on antidepressant medications at the time of testing may suggest that a “mild depression” level characterized the majority of the participants at the time of testing.

Finally, an alternative as well as theoretically interesting account for the present null finding may be based on current notions about the circuitry underlying processing of emotional information; that is, neuroscience studies indicate that the amygdala is a key structure of the emotional/social brain but its activity modulates—and is in turn reciprocally modulated by—cortical structures like the prefrontal medial cortex and the anterior cingulate gyrus (Price and Drevets, 2011). Hence, an exchange of signals regarding external stimuli and the emotional response of the individual may depend on a consensus between evaluations of the stimuli performed by all three regions simultaneously. However, in the present experiment the low-passed emotional content of hybrid pictures may be strongly processed by subcortical structures like the amygdala and, at the same time, only weakly engage cortical areas, which would preferentially elaborate the high-pass information. Given that the high-pass information delivers a neutral expression, which is incongruent with the low-passed emotional content, the amygdala response may lack essential re-entrant or feedback information that is necessary to trigger a full-blown response. In other words, a hyperactive response of the amygdala may actually depend on congruent information being processed by the cortical system and, in turn, exaggerated responses to negative stimuli may be related to the status of the whole connected emotional system and not by a component alone. Future studies should compare, for the same depressed participants, social judgments and pupillary responses to emotional stimuli that are presented in an “explicit” manner and can be consciously apprehended vs. the same type of emotional stimuli as viewed in an “implicit” condition (as that used in the present study).

The present study is not without limitations. First, our clinical sample (currently and previously depressed) was characterized by a mild to moderate depression level as indicated by the BDI–II. It might be argued that using a clinically administered measure for depression, such as the Montgomery–Åsberg Depression Rating Scale (MADRS; Montgomery and Åsberg, 1979), would have given a more sensitive assessment of actual depression severity. On the other hand, the grouping of subjects in our study (currently depressed, recovered previously depressed, and never depressed) was made according to the information given in the clinical SCID-I interview and the *DSM–IV–TR* criteria. None of the subjects was hospitalized. It is fairly well-established that major depression is often not adequately recognized in primary care (Kamphuis et al., 2012). Thus, we accept this as a reasonable explanation of why so few MDD patients were medicated. Future studies on clinical samples are indicated to test the generalizability of our findings to inpatients and more severely depressed individuals.

### Conflict of interest statement

The authors declare that the research was conducted in the absence of any commercial or financial relationships that could be construed as a potential conflict of interest.
